# A Combination of Novel Nucleic Acid Cross-Linking Dye and Recombinase-Aided Amplification for the Rapid Detection of Viable *Salmonella* in Milk

**DOI:** 10.3390/foods11152375

**Published:** 2022-08-08

**Authors:** Xiaoyan Feng, Donggen Zhou, Bei Gan, Guoyang Xie, Hengyi Xu

**Affiliations:** 1 State Key Laboratory of Food Science and Technology, Nanchang University, Nanchang 330047, China; 2 Ningbo International Travel Healthcare Center (Ningbo Customs Port Outpatient Department), Ningbo 315010, China; 3 Institute for Testing of Industrial Products of Jiangxi General Institute of Testing and Certification, Nanchang 330047, China

**Keywords:** *Salmonella*, thiazole orange monoazide, recombinase-aided amplification, milk

## Abstract

*Salmonella*, as an important foodborne pathogen, can cause various diseases, such as severe enteritis. In recent years, various types of nucleicacid-intercalating dyes have been utilized to detect viable *Salmonella*. However, in principle, the performance of existing nucleic acid dyes is limited because they depend on the integrity of cell membrane. Herein, based on the metabolic activity of bacteria, a novel DNA dye called thiazole orange monoazide (TOMA) was introduced to block the DNA from dead bacteria. Recombinase-aided amplification (RAA) was then performed to detect viable *Salmonella* in samples. In this study, the permeability of TOMA to the cell membrane of *Salmonella* was evaluated via confocal laser scanning microscopy and fluorescence emission spectrometry. The limit of detection (LOD) of the TOMA–RAA method was 2.0 × 10^4^ CFU/mL in pure culture. The feasibility of the TOMA–RAA method in detecting *Salmonella* was assessed in spiked milk. The LOD for *Salmonella* was 3.5 × 10^2^ CFU/mL after 3 h of enrichment and 3.5 × 10^0^ CFU/mL after 5 h of enrichment. The proposed TOMA–RAA assay has great potential to be applied to accurately detect and monitor foodborne pathogens in milk and its byproducts.

## 1. Introduction

Nowadays, consumer’s demand for milk and milk products has been increasing because they are rich in nutrients that meet the requirements of human health [1]. However, milk, with a neutral pH and high moisture and protein contents, can induce the growth of microorganisms [2]. Although dairy industries have implemented measures to eliminate these pathogens, such as pasteurization and ultra-high temperature instantaneous sterilization, outbreaks of foodborne diseases due to the consumption of contaminated milk still occur [3,4]. *Salmonella* is the most common pathogen in contaminated milk [5,6,7], causing harm to human health, such as fever, diarrhea, and enteritis [8,9]. To face these threats, an accurate and specific method for detecting *Salmonella* in milk is imperatively required.

Conventional culture-based methods are incompetent for rapid analysis for the presence of *Salmonella* due to their disadvantages, including being time-consuming, laborious, and inaccurate [10,11]. In recent years, isothermal nucleic acid amplification techniques, such as rolling circle amplification [12,13], and loop-mediated isothermal amplification [14,15], have emerged and been applied for bacterial pathogen detection due to the advantage of a constant temperature. Among these, on account of the convenience and a shorter detection time, the performance of recombinase-aided amplification (RAA) technology is superior to that of other methods and has been used in conjunction with several methods for pathogen identification [16,17,18]. The RAA technology works in the presence of adenosine triphosphate. First, oligonucleotides pair with the recombinase protein UvsX to form nucleoprotein complexes, which interrogate double-stranded DNA (dsDNA) to seek their homologous sequences. Then, the replaced single-stranded DNA forms a D-loop structure and is stabilized by a single-stranded binding (SSB) protein to prevent the ejection of the inserted primer by branch migration. Elongation is then activated with the assistance of DNA polymerase. Finally, the cyclic repetition of this process is implemented to achieve the exponential amplification of dsDNA in vitro [19,20]. Hence, the RAA assay is applicable for field tests due to its rapid analysis, low cost, and high specificity [21].

**Figure 1:**
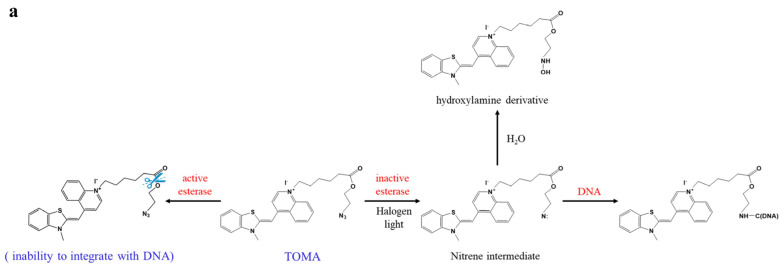

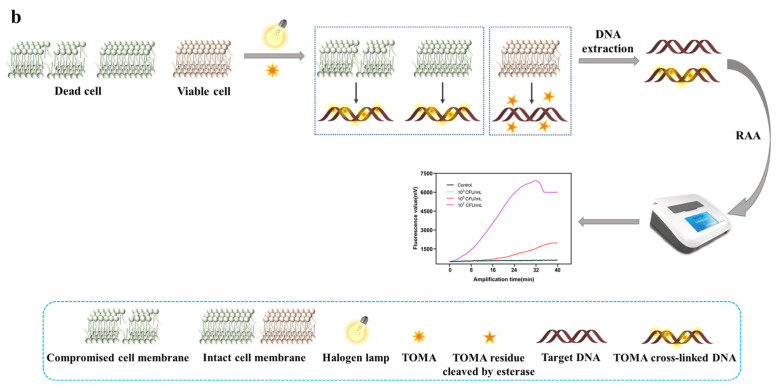
Working principle of the TOMA–RAA method for viable *Salmonella* detection. (**a**) The structure of the TOMA molecule and the process of TOMA integrating with DNA; (**b**) the detection process of the TOMA–RAA method for the specific detection of viable *Salmonella*.

It is true that RAA technology, indeed, fails to accurately detect viable bacteria. Accordingly, various types of DNA dyes, such as ethidium monoazide (EMA) [22,23] and propidium monoazide (PMA) [24,25], have been used to eliminate the signals of dead cells. These DNA-intercalating dyes can be utilized in distinguishing dead cells from viable cells based on the criterion of whether the cell membrane is disrupted. Nocker and Camper [26] argued that considering culturability and cell membrane integrity as the criterion for bacteria viability is inaccurate and conservative. Therefore, they introduced the novel concept of “activity-labile compounds”, suggesting that metabolic activity could be used as a criterion for bacterial viability. This compound consists of three parts: a DNA-intercalating molecule, a cross-linkable molecule, and a linker cleaved by esterase, which is a ubiquitous enzyme that is widely found in cells with an active metabolism. Based on the present study, a novel DNA-intercalating dye called thiazole orange monoazide (TOMA) was introduced, which is a combination of thiazole orange (TO) and an azide group linked by an ester bond. The successful application of TOMA was attributed to the ability of the TO dye to permeate all cells [27] and the ability of the azide group to integrate with DNA. As shown in Figure 1a, in viable cells active esterase cleaves the ester bond, and the azide group is removed from the TOMA molecule. Thus, the TOMA molecule fails to covalently cross-link with DNA in viable cells due to the absence of the azide group. On the contrary, in dead cells lacking active esterase, the ester bond fails to be cleaved, resulting in a TOMA molecule that can integrate with DNA. At present, some studies have determined the properties of TOMA and explored its potential applications in the detection of bacterial pathogens [28,29].

Herein, a TOMA–RAA method was established as a novel, rapid, and accurate method for detecting viable *Salmonella* in milk samples. The specific primers and probe were used to guarantee the specificity of this strategy. Under optimal conditions, the target bacteria were subjected to a TOMA treatment prior to the RAA assay to remove the signals of dead cells. Furthermore, the properties of TOMA, as well as the feasibility and sensitivity of this TOMA–RAA method for *Salmonella* detection in skim milk samples, were evaluated in this study. All in all, this novel method provides a new research direction for the detection of viable pathogenic bacteria.

## 2. Materials and Methods

### 2.1. Reagents and Materials

Dimethyl sulfoxide (DMSO) was obtained from Shanghai Yuanye Bio-Technology Co., Ltd. (Shanghai, China). Proteinase K was bought from Tiangen Biotech Co., Ltd. (Beijing, China). Antifade Mounting Medium was bought from Boster Biological Technology Co., Ltd. Paraformaldehyde fix solution at a concentration of 4% was obtained from Servicebio Technology Co., Ltd. (Wuhan, China). An RAA fluorescence kit was obtained from Jiangsu Qitian Gene Biotechnology Co., Ltd. (Wuxi, China). A Wizard^®^ Magnetic DNA Purification System for Food was purchased from Promega Biotech Co., Ltd. (Madison, WI, USA). All primers and probes were synthesized by Sangon Biotech (Shanghai, China). Skim milk was purchased at the local supermarket (Rainbow, China). TOMA was provided by Ningbo International Travel Healthcare Center (Ningbo Customs Port Outpatient Department). The final concentration of TOMA dissolved in 20% DMSO was 1 mg/mL. It was stored at −20 °C in the dark for further use.

### 2.2. Apparatus

A QT-RAA-B6100 constant temperature oscillometer microplate (Jiangsu Qitian Gene Bio-Technology Co. Ltd., Wuxi, China) was used to mix these reaction units thoroughly. The relationship between the fluorescence value and the amplification time was obtained by the QT-RAA-F1620 fluorescence detector (Jiangsu Qitian Gene Bio-Technology Co. Ltd., Wuxi, China). The fluorescence images after the TOMA treatment were observed by confocal laser scanning microscopy (CLSM) (Leica TCS SP8, Solms, Germany), and the fluorescence spectrum was obtained by a microplate reader (Varioskan LUX, Thermo Fisher Scientific, Vantaa, Finland).

### 2.3. Bacterial Strains and Culture Condition

*Salmonella* enterica subsp. enterica serovar Enteritidis ATCC13076 was chosen as an experimental strain for RAA in this study. All bacterial strains were cultured in Luria–Bertani (LB) medium overnight at 37 °C with shaking at 180 rpm. Bacterial pellets were collected after centrifugating at 9600× *g* for 5 min and resuspended in sterile phosphate buffer saline (PBS, 0.01 M, pH 7.40). Bacterial suspensions were 10-fold serially diluted to the appropriate concentrations (ranging from 10^2^ to 10^4^ CFU/mL), then grown on an LB plate for 24 h to obtain the original concentration of bacterial strains.

### 2.4. RAA Reaction Program

The oligonucleotide sequences of the primers and probe are shown in Table 1. The RAA assay was performed following an RAA fluorescence kit. Each reaction unit was performed in a total volume of 50 μL, containing 25 μL of rehydration buffer, 12.7 μL of deionized water, 2.1 μL of each primer (10 μM), 0.6 μL of fluorescent probe (10 μM), 2.5 μL of magnesium acetate (280 mM), and 5 μL of DNA template, then added to the freeze-dried powder (800 ng/μL SSB, 120 ng/μL UvsX, and 30 ng/μL DNA polymerase). Reaction units were placed in a QT-RAA-B6100 microplate, thoroughly mixed, then transferred to a QT-RAA-F1620 detector to obtain the curve of the fluorescence value and the amplification time. This RAA reaction was performed at 39 °C within 40 min.

### 2.5. Verification of the Permeability of the Cell Membrane to TOMA

The permeability of TOMA dye was evaluated using CLSM and fluorescence intensity. A suspension of *Salmonella* cells at a concentration of 10^8^ CFU/mL was used to prepare viable and dead cells, of which dead cells were obtained by heating at 80 °C for 20 min and cooling at 4 °C for 5 min. The TOMA solution was added into these bacterial suspensions and incubated for 20 min in the dark. To remove excess TOMA dye, these samples were centrifugated at 9600× *g* for 5 min prior to washing with PBS. Then, these treated samples were fixed in 4% paraformaldehyde. Finally, 10 μL of prepared samples containing anti-quenching agent were dripped onto clean glass slides and observed by CLSM. Meanwhile, another 100 μL of prepared samples were transferred to a microplate reader to obtain the fluorescence spectrum.

### 2.6. Parameter Optimization

Viable and dead *Salmonella* suspensions at 10^6^ CFU/mL were prepared to optimize the experimental parameters, including the TOMA concentration and exposure time. All samples were treated as follows: TOMA solution was added to prepared samples and incubated for 20 min in the dark. Then, these mixtures were placed on the ice and exposed for a certain time under a 500 W halogen light source with occasional shaking to ensure uniform light. To remove excess TOMA, these samples were centrifuged at 9600× *g* for 5 min, and cell pellets were washed with PBS. Genomic DNA was extracted for the RAA assay. For the TOMA concentration optimization, 0, 5, 10, 20, 30, and 50 μg/mL of TOMA solution were tested. Exposure times including 0, 5, 10, 15, 20 and 30 min were used to optimize the method.

### 2.7. Sensitivity Analysis of this TOMA–RAA Method for Viable *Salmonella* in Pure Culture

To determine the limit of detection (LOD) of the TOMA–RAA method for viable *Salmonella* in pure culture, a 10-fold serial dilution of *Salmonella* culture from 2 × 10^3^ to 2 × 10^7^ CFU/mL was subjected to TOMA treatment, then target DNA was obtained by following the manufacturer’s instructions for the Wizard^®^ Magnetic DNA Purification System for Food. Finally, the obtained DNA templates were used for the RAA assay, while sterile deionized water replacing the target DNA template was used for the negative control. All experimental procedures are shown in Figure 1b.

### 2.8. Recovery Assessment of Viable Salmonella by the TOMA–RAA Method

We prepared various bacterial suspensions to assess the accuracy of the proposed method, including three groups: (1) 10^7^ CFU/mL of viable cells and 10^6^ CFU/mL of dead cells, (2) 10^6^ CFU/mL of viable cells and 10^6^ CFU/mL of dead cells, and (3) 10^5^ CFU/mL of viable cells and 10^6^ of CFU/mL dead cells. Subsequently, genomic DNA was extracted and subjected to the TOMA–RAA assay to obtain fluorescence curves.

### 2.9. Assessment of the TOMA–RAA Method Using Artificially Spiked Skim Milk

Packaged skim milk was disturbed on an LB plate at 37 °C for 24 h to verify that it was negative for pathogens. The 10-fold serially diluted *Salmonella* suspensions were prepared. To prepare the spiked milk samples, 100 μL of *Salmonella* suspension was blended with 900 μL of skim milk to obtain concentrations from 2.1 × 10^2^ to 2.1 × 10^7^ CFU/mL. These spiked mixtures were centrifugated at 9600× *g* for 5 min and resuspended in 500 μL of PBS. After TOMA treatment, proteinase K (20 mg/mL) was added to eliminate protein from milk samples, which may affect the extraction of DNA, and target DNA was extracted for the RAA assay to obtain the LOD of this established method in spiked skim milk.

### 2.10. Data Analysis

All data were obtained by RAA-F1620 software matching with an RAA-F1620 fluorescence detector, and calculations were conducted using Excel software. The pictures were plotted with GraphPad Prism 8 (GraphPad Prism software Inc., San Diego, CA, USA).

## 3. Results and Discussion

### 3.1. Permeability of Cell Membrane to TOMA

**Figure 2:**
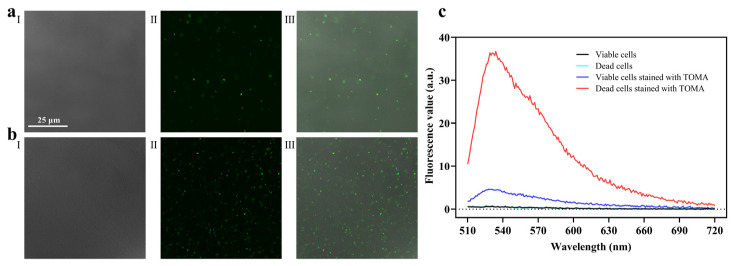
Characterization analysis of the permeability of TOMA to the cell membrane. CLSM images of (**a**) viable *Salmonella* and (**b**) dead *Salmonella*: (**I**) light image with no stain; (**II**) with TOMA treatment; (**III**) merged image; (**c**) Fluorescence emission spectrum (the excitation wavelength was 488 nm) of *Salmonella* after the TOMA treatment.

As an asymmetric cyanine dye, TO can freely penetrate into all cells [30], but it does not produce fluorescence in aqueous solution. Once TO integrates with nucleic acids, it generates a remarkable fluorescence enhancement on account of the restriction of intramolecular rotations between benzothiazole and quinoline heterocycles [31,32]. Accordingly, TO is often used as a fluorescent probe for nucleic acid detection [33]. In this study, the permeability of cell membrane to TOMA was proven because the presence of the TO structure integrated with nucleic acids showed green florescence. As shown in Figure 2a, there was no fluorescence in both viable and dead *Salmonella* without TOMA treatment at 488 nm (excitation wavelength), whereas viable and dead *Salmonella* with TOMA treatment produced a strong green fluorescence. As further proof, the fluorescence spectrum was obtained using a microplate reader (Figure 2b). The fluorescence value of both viable and dead cells at 530 nm was close to zero. After the TOMA treatment, the fluorescence value of viable cells reached 4.61, whereas that of dead cells reached 35.85, indicating that TOMA could enter both viable and dead cells. These results demonstrated that TOMA is permeable to the cell membrane. Thus, it can be used as a novel dye for pathogen detection.

### 3.2. Optimization of Parameters

The azide group in the TOMA molecule is the same as that in PMA and EMA. Under a strong light source, the free nitrogen of the azide group is converted to a highly active azene, which cross-links with DNA to form a stable covalent compound in situ to inhibit DNA amplification, whereas unbound TOMA binds with water to form hydroxylamine derivatives [28]. The reaction process is shown in Figure 1a. Some parameters should be optimized to achieve the best performance of the TOMA treatment.

**Figure 3:**
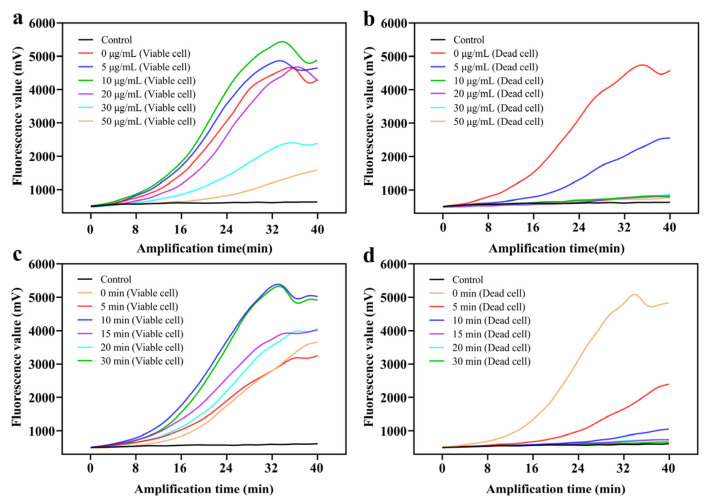
Optimization of reaction conditions. The fluorescence curve of (**a**) viable cells and (**b**) heat-killed dead cells subjected to TOMA treatment at 0, 5, 10, 20, 30, and 50 μg/mL; the fluorescence curve of (**c**) viable cells and (**d**) dead cells after exposure for 0, 5, 10, 15, 20, and 30 min.

The accuracy for detecting viable bacteria was found to be affected by the TOMA concentration. As an important parameter, low concentrations of TOMA caused a false-positive result because dead bacteria interfered with the RAA assay. By comparison, excessive TOMA resulted in a false-negative result due to TOMA cross-linking with DNA from viable bacteria. In Figure 3a, as the TOMA concentration increased from 0 to 30 μg/mL in viable cells, no remarkable change in inhibition after the RAA assay was observed. When the TOMA concentration was ≥30 μg/mL, the inhibition was obvious, probably because the excessive TOMA integrated with DNA and DNA amplification was inhibited. In dead cells, as TOMA increased from 0 to 50 μg/mL, DNA amplification was remarkably inhibited (Figure 3b). When the TOMA concentration was 10 μg/mL, the inhibition of DNA amplification reached a plateau. Therefore, 10 μg/mL was chosen as the optimal TOMA concentration to ensure that no effect would be exerted on viable bacteria.

The exposure time, another important parameter, had a great impact on the accuracy of the detection of viable cells. All optimized results are summarized in Figure 3c,d. No obvious inhibition of viable cells after the RAA assay was observed with increases in exposure time (Figure 3c). However, the inhibition of dead cells had no remarkable change when the exposure time was over 15 min (Figure 3d). Finally, 15 min was used as the optimal exposure time.

### 3.3. Specificity and Performance of this TOMA–RAA Method in Pure Culture

The primer and probe were the crucial factors that needed to be considered to ensure the specificity of the TOMA–RAA method. Hence, the probe and primer were designed on the basis of the specific *invA* gene of *Salmonella* [34,35] (Table 1). Eighteen bacterial strains were used to verify the specificity of the probe and primer. A fresh bacterial culture with a density of 10^8^ CFU/mL was used for DNA extraction, then the RAA assay was performed. As shown in Table 2, the RAA assay for four target strains showed positive results, while nontarget strains showed negative results, indicating that the established TOMA–RAA method had a high specificity for different serovars of *Salmonella* strains.

**Figure 4:**
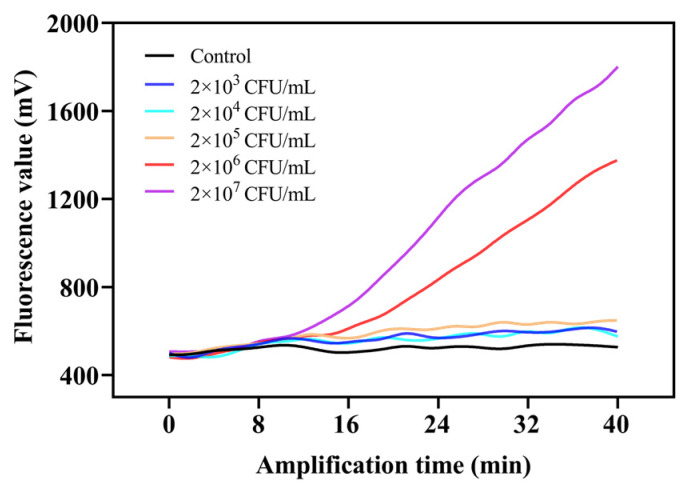
The LOD for viable *Salmonella* in pure culture by the TOMA–RAA method.

The LOD of the TOMA–RAA method was determined in pure culture. Under optimal conditions, diluted *Salmonella* suspensions were subjected to the TOMA–RAA method. The RAA reaction was accomplished in 40 min at 39 °C. Subsequently, fluorescence curves of *Salmonella* suspensions ranging from 2 × 10^3^ to 2 × 10^7^ CFU/mL were obtained. The LOD value was defined as the lowest concentration of *Salmonella* suspension whose fluorescence signal was higher than that of the negative control group. As shown in Figure 4, the LOD of the TOMA–RAA method for *Salmonella* was 2 × 10^4^ CFU/mL in pure culture.

### 3.4. Sensitivity of this Proposed Strategy in Spiked Skim Milk

**Figure 5:**
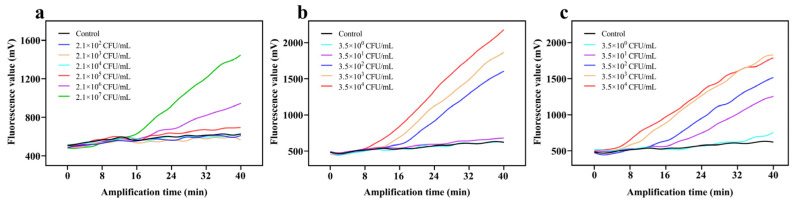
The sensitivity analysis of the TOMA–RAA method for the detection of viable *Salmonella* in skim milk. (**a**) Fluorescence curve with different concentrations of *Salmonella* in pure culture; (**b**) Fluorescence curve after 3 h of enrichment; (**c**) the fluorescence curve after 5 h of enrichment.

Spiked skim milk was prepared as a real matrix to assess the practicability of the proposed TOMA–RAA method. As shown in Figure 5a, the LOD of the TOMA–RAA method was 2.1 × 10^5^ CFU/mL in spiked skim milk. However, the level of *Salmonella* in the milk sample might be lower. Accordingly, the sensitivity of the TOMA–RAA method for *Salmonella* detection after pre-enrichment in spiked skim milk was evaluated. *Salmonella* suspensions ranging from 3.5 × 10^0^ to 3.5 × 10^4^ CFU/mL were added to skim milk at 3 and 5 h of enrichment time (Figure 5b,c). After 3 h of enrichment, the lowest concentration of *Salmonella* detected by the TOMA–RAA method was 3.5 × 10^2^ CFU/mL (Figure 5b). After 5 h of enrichment, the LOD of the TOMA–RAA method for *Salmonella* was 3.5 × 10^0^ CFU/mL (Figure 5c). These results suggested that this method may have good performance in detecting *Salmonella* in a food matrix.

### 3.5. The Recovery Assessment for the Proposed TOMA–RAA Method

**Figure 6:**
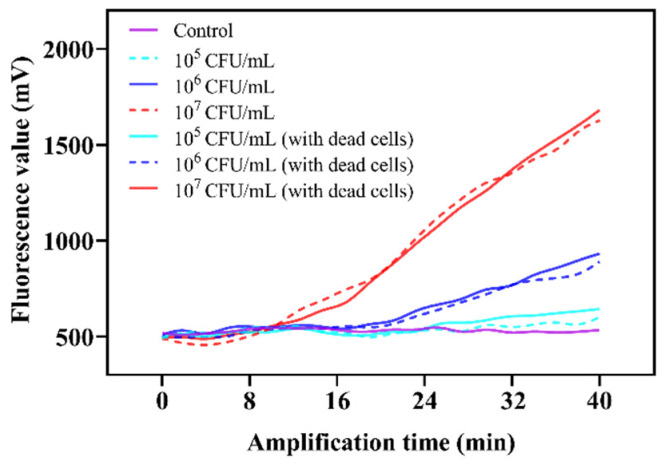
Evaluation of the anti-interference ability for dead *Salmonella* based on the TOMA–RAA method. The concertation of dead *Salmonella* was 10^6^ CFU/mL in PBS.

In practice, there might be the presence of dead bacteria in food samples. Hence, different ratios of viable and dead bacteria were used to assess the anti-interference ability of the proposed TOMA–RAA method for the accurate detection of viable *Salmonella*. The results of the recovery assessment are shown in Figure 6, and there was no influence on the detection of viable *Salmonella* by the presence or absence of different concentrations of dead *Salmonella*, indicating that this method has a strong anti-interference ability and can be used to detect viable *Salmonella*.

## 4. Conclusions

A new DNA-intercalating dye, namely, TOMA, was introduced and combined with the RAA assay for detecting viable *Salmonella*. In the first place, CLSM and the fluorescence emission spectrum confirmed that TOMA was permeable to the cell membrane. In optimized conditions, the LOD of viable *Salmonella* was 2 × 10^4^ CFU/mL in pure culture. Moreover, the TOMA–RAA method was applied to detect viable *Salmonella* in spiked skim milk. The LOD was 3.5 × 10^2^ CFU/mL after 3 h of enrichment, while it was 3.5 × 10^0^ CFU/mL after 5 h of enrichment. The presence of different concentrations of dead *Salmonella* did not influence the detection of viable *Salmonella*. In a word, the principle of this TOMA dye depends on the metabolic activity of viable bacteria. It was demonstrated to be more accurate than existing DNA dyes. This method could be developed as an accurate, qualitative, and specific method for detecting viable *Salmonella* without interference. Thus, it provides a technical reference for the detection of viable pathogenic microorganisms in contaminated milk.

## Figures and Tables

**Table 1:** The information of primer and probe in this study.

Primer/Probe	Sequence (5′-3′)	Amplicon Length
*invA*-Forward	ATTGGCGATAGCCTGGCRGTGGGTTTTGTTGTC	
*invA*-Reverse	TACCGGGCATACCATCCAGAGAAAAWCGDGCCGC	133 bp
Probe	CTCKATTGTCACKGTGGTYCAGTTTATCG(FAM-dT)T(THF)T(BHQ-dT)ACCAAAGGTTCA	

Footnote: FAM: 6-Carboxyfluorescein; THF: tetrahydrofuran; BHQ: black hole quencher; phosphate: 3′ phosphate to block elongation.

**Table 2:** Bacterial strains used in this study and the results of the RAA assay.

Bacterial Strains	Strain ID ^a^	RAA Results ^b^
*Salmonella* Enteritidis	ATCC 13076	+
*Salmonella* Typhimurium	ATCC 14028	+
*Salmonella* Abaetetuba	ATCC 35640	+
*Salmonella* Paratyphi A	ATCC 9150	+
*Klebsiella pneumoniae*	ATCC 700603	-
*Staphylococcus aureus*	CMCC 26001	-
	ATCC 25923	-
Methicillin-resistant *Staphylococcus aureus*	NCTC 12493	-
*Bacillus stearothermophilus*	ATCC 7953	-
*Escherichia coli*	CMCC 44102	-
	ATCC 25922	-
*Pseudomonas aeruginosa*	CMCC 10104	-
*Deinococcus radiophilus*	ATCC 27603	-
*Shigella sonnei*	ATCC 25931	-
*Cronobacter malonaticus*	CMCC 45402	-
*Cronobacter sakazakii*	ATCC 29544	-
*Listeria monocytogenes*	ATCC 13932	-
*Listeria innocua*	NCTC 11288	-

^a^ ATCC = American Type Culture Collection, USA; CMCC = China Medical Culture Collection; JX-CDC = Jiang Xi Province Center for Disease Control and Prevention, China; NCTC = National Collection of Type Cultures, United Kingdom. ^b^ “+” represents positive result, “-” represents negative result.

## Data Availability

The data presented in this study are available on request from the corresponding author.
